# Swab test in biological fluids as predictor of COVID-19 transmission risk during surgery: a prospective cross-sectional study from an Italian COVID center

**DOI:** 10.1186/s12893-022-01571-6

**Published:** 2022-03-30

**Authors:** N. Fabbri, A. Pesce, A. Ussia, F. D’Urbano, S. Pizzicotti, S. Greco, C. V. Feo

**Affiliations:** 1 General Surgery Unit, Azienda USL Di Ferrara, Via Valle Oppio, 2, Ferrara, Italy; 2 Department of Translational Medicine, Azienda Ospedaliero-Universitaria Di Ferrara, Ferrara, Italy; 3 Biochemical Analysis Laboratory - Clinics and Microbiology, Azienda Ospedaliero-Universitaria Di Ferrara, Ferrara, Italy; 4 Department of Medical Sciences, Università Di Ferrara, Ferrara, Italy

**Keywords:** COVID-19, Surgery, Pandemics, Operating rooms

## Abstract

**Background:**

The contamination of body fluids by Severe Acute Respiratory Syndrome Coronavirus 2 during surgery is current matter of debate in the scientific literature concerning CoronaVIrus Disease 2019. Surgical guidelines were published during the first wave of the COVID-19 pandemic and recommended to avoid laparoscopic surgery as much as possible, in fear that the chimney effect of high flow intraperitoneal gas escape during, and after, the procedure would increase the risk of viral transmission.

**Aim:**

The aim of this study was to evaluate the possibility of SARS-CoV-2 transmission during surgery by searching for viral RNA in serial samplings of biological liquids.

**Methods:**

This is a single center prospective cross-sectional study. We used a real-time reverse transcriptase (RT) polymerase chain reaction (PCR) test to perform swab tests for the qualitative detection of nucleic acid from SARS-CoV-2 in abdominal fluids, during emergency surgery and on the first post-operative day. In the case of thoracic surgery, we performed a swab test of pleural fluids during chest drainage placement as well as on the first post-operative day.

**Results:**

A total of 20 samples were obtained: 5 from pleural fluids, 13 from peritoneal fluids and two from biliary fluid. All 20 swabs performed from biological fluids resulted negative for SARS-CoV-2 RNA detection.

**Conclusion:**

To date, there is no scientific evidence of possible contagion by laparoscopic aerosolization of SARS-CoV-2, neither is certain whether the virus is effectively present in biological fluids.

**Supplementary Information:**

The online version contains supplementary material available at 10.1186/s12893-022-01571-6.

## Background

The possible contamination of body fluids by Severe Acute Respiratory Syndrome Coronavirus 2 (SARS-CoV-2) during surgery is a recent topic of discussion in the scientific literature concerning COronaVIrus Disease 2019 (COVID-19) [1].

Previous studies on different viruses reported that viruses like hepatitis B virus, HIV, and HPV were previously detected in aerosolizations of biological liquids, during laparoscopic surgery [2–5].

Based on these data, the use of personal protective equipment (PPE) was advocated by national and international guidelines as the best method to reduce the risk of contamination in theatre staff, especially during aerosol-generating procedures.

In May 2020, the European Society of Coloproctology released a joint statement with the European Association of Endoscopic Surgery (EAES) and the *Society of American *Gastrointestinal* and Endoscopic Surgeons* (SAGES) recommending pre-operatory COVID-19 testing for all surgical patients, intubation and extubation in negative pressure rooms, and appropriate filtration and ventilation of the operating rooms in case of suspected or confirmed COVID-19 patients [6]. Surgical guidelines were published during the first wave of the pandemic and recommended to avoid laparoscopic surgery as much as possible, in fear that the chimney effect of high flow intraperitoneal gas escape during and after the procedure would increase the risk of viral transmission. However, recent systematic reviews investigated the role of surgical aerosol and COVID-19 infection, concluding that, to date, still no evidence of a link between the two factors exists [7, 8].

Although the absence of the virus in laparoscopic fumes seems to be demonstrated, there are still insufficient data concerning the risk of contagion from biological fluids during surgery. Some considerations for and against the possibility of a viral translocation exist: first, it is widely known that peritoneal membranes have a maximum pore diameter ranging from 20 to 40 nm, while the diameter of SARS-CoV-2 virion measures approximately 50–200 nm. For this reason, it is not possible to exclude a theoretical transmigration of the virus across the barrier represented by the peritoneal membrane, especially in case of increased permeability (inflammation) or damage.

Furthermore, the cell membrane protein angiotensin-converting enzyme-2 (ACE-2) is the key for receptor-mediated cell entry of SARS-CoV-2. This protein is expressed in pneumocytes (type II alveolar cells) as well as in the gastrointestinal tract (in particular in ileal and colonic enterocytes) and it may represent “the access door” through the peritoneal membrane during surgery, both open and laparoscopic [9].

Despite the theoretical risk of contamination, only few case reports confirmed the presence of SARS-CoV-2 in abdominal fluids [10–12], while some researchers also reported the viral involvement of pleural fluids [13–16].

The aim of this study was to evaluate the possibility of viral transmission of SARS-CoV-2 during surgery by searching for viral RNA in serial samplings of biological liquids.

## Material and methods

This is a single center prospective cross-sectional study, performed in a hospital serving a wide and low densely populated rural area in the province of Ferrara, Italy. We used a real time reverse transcriptase (RT) polymerase chain reaction (PCR) test to perform swab tests for qualitative detection of nucleic acid from SARS-CoV-2 (Alinity m SARS-CoV-2 assay, Abbott, USA). For abdominal fluids, a first abdominal swab sample was performed at the time of abdominal incision, while a second swab sample was collected at the end of the surgical operation, before washing the abdominal cavity with saline solution. In case of cholecystectomy, a biliary swab test was also collected. On the first post-operative day, a swab sample of fluid from the abdominal drainage (if placed) was collected together with a naso-pharyngeal swab. In thoracic surgery, we performed a swab test of pleural fluids during chest tube placement as well as on the first post-operative day.

In order to review the literature on this topic, we used the advanced searching function of PubMed library. The key words used were the following: “Peritoneal Fluid”, “Pleural Fluid”, “COVID” and “SARS-CoV-2”. The research was limited to studies written in English, including adult patients (older than 18 years): we found a total of 13 articles concerning abdominal fluids and 4 about thoracic fluids.

## Results

From February 2020 to May 2021, a total of 8 COVID-19 patients underwent emergency surgery (Additional file 1: Table S1); 5 were females and 3 males, while the mean age was 78 years (range 44–92 years); 2 patients were operated during the first wave of the pandemic, 2 more during the second wave, and 4 during the third wave. The following surgical procedures were performed:

- 3 chest tube placements for pneumothorax (one male and two females)
- 2 open sigmoid Hartmann resections for perforated diverticulitis (both females)
- 1 exploratory laparotomy for massive bowel infarction (male)
- 1 laparoscopic cholecystectomy (male)
- 1 open cholecystectomy (male)

A total of 20 samples were obtained in 8 patients: 5 from pleural fluids (all in patients with pneumothorax), 13 from peritoneal fluids, and 2 from biliary fluid. 6 out of 8 patients had a diagnosis of COVID-19 related pneumonia, while two of them did not have lung involvement by SARS CoV-2. All subjects were tested positive for SARS-CoV-2 RNA detection through naso-pharyngeal swab. 4 patients died during hospitalization, those with pneumothorax and the one with massive bowel infarction. All 20 swabs performed from biological fluids resulted negative for SARS-CoV-2 RNA detection.

## Discussion

This study focused on evaluating the presence of SARS-CoV-2 RNA in a total of 20 peritoneal and pleural fluid samples from 8 COVID-19 patients (5 females and 3 males) who underwent emergency surgery over a period of 15 months.

Based on our single center experience, we could not demonstrate the presence of the virus in any of the samples. Overall, we investigated a total of 8 patients with positive SARS-CoV-2 RNA naso-pharyngeal swab undergoing emergency surgery. With the limits of the small size of sampling, we registered a prevalence of females (5 out 8 patients, 62.5%) and a greater number of swabs performed from peritoneal fluid (13 against 5 taken from pleural fluid and 2 from bile). 4 out of 8 patients died during hospitalization due to the progressive deterioration of their clinical condition.

In a recent article, some Authors performed SARS-CoV-2 RT-PCR assay from the peritoneal effluent after dialysis of a symptomatic COVID-19 patient: the assay was performed twice after 6-h duration of dwells without centrifugation, but it resulted negative in both samples [17]. There are only few cases reporting positive peritoneal swab test (RT-PCR assays), but in these cases, patients were often affected by conditions that could theoretically favor the diffusion of fluids across the peritoneal membranes, such as kidney transplant in a patient with hepatic cirrhosis [12], peritoneal dialysis [18], and bowel contamination during emergency surgery [11].

Some authors also reported the presence of SARS-CoV-2 RNA within the intestinal tissues during upper gastrointestinal surgery [19] or inside the appendix and its lymph node [20]. However, none of these studies highlighted the presence of the virus in the peritoneal fluid. Spontaneous pneumothorax is a possible complication of COVID-19 pneumonia and it may occur even in patients not treated with mechanical ventilation or high-flow oxygen therapy, even if such reports seem to be anecdotic [21]. With respect to patients with SARS-CoV-2 pneumonia, the prevalence of pleural effusion can be as high as 14% [14]. However, we found only 4 articles concerning the involvement of pleural fluids by SARS-CoV-2 (all subjects were tested positive for RNA detection through RT-PCR assay): specifically, a 61-year-old man who underwent kidney transplant [13], a 71-year-old Afro-American man with no comorbidities [14], a 68-year-old Chinese man with lung cancer and an Italian 72-year-old man with only hypertension in his clinical history [16].

The presence of SARS-CoV-2 RNA in other tissues, such as sputum and stool, was already demonstrated [22].

Tests of feces with molecular swabs allowed to detect SARS-CoV-2 RNA in a percentage of infected patients ranging from one third to one half, suggesting the potential local viral translocation in particular favorable conditions [23]. To date, based on the available scientific data, the probability of contagion by laparoscopic aerosolization of the virus is low and even lower is the evidence documenting the presence of SARS-CoV-2 in biological fluids [10–12], especially in the case of thoracic surgery.

Although aerosolization of blood borne viruses was already shown to occurr during laparoscopy, several reports did not demonstrate the presence of SARS-CoV-2 RNA in the peritoneal fluid of COVID-19 patients [1, 24–28].

Our review of literature showed that most studies are in favor of the absence of SARS-CoV-2 RNA in biological fluids (peritoneal and pleural) when swab tests are performed during surgery, while positive viral reports are limited to patients with immune system compromission (patients with organ transplantation or with cancer) or with peritoneal lymphatic drainage illnesses (e.g. cirrhotic patients).

We tried to hypothesize some possible explanations of these findings. First of all, the virus may be present but undetectable through the available tests. The sensitivity of molecular swabs was already evaluated in some studies that cast doubts on possible false negatives results, due to sampling problems or to thermal damage of the swabs: this could happen in case of patients with low viral load [29, 30]. Furthermore, the laboratory method for the diagnosis of SARS-CoV-2 is usually certified as qualitative and, therefore, the result of molecular swab is either negative or positive, depending on the number of cycles of viral replication. The chemo-physical process underlying this kind of test is much more complicated, indeed, and the result of molecular swabs does not depend only on exceeding a threshold value but also on the extent of the excess.

Moreover, it shall be considered that a “dubious” result (a weak positivity or negativity) can also be caused by factors that are usually unrelated to the virus itself or to its load, such as the method of storage or transportation or sampling. The difficulties encountered in the interpretation of some swab tests results can be partially overcome by recognizing such intrinsic disadvantages of the process itself and, at the same time, this goes to explain why some patients can meet discordant results in case of samples from different body fluids. Finally, it is to remind that the only swabs currently certified are those taken from the oropharynx or from bronchoalveolar lavage [9].

The second possibility is about the inactivity of the virus, that may be present (either detectable or not). To date, no certified case of contagion between patient and operator during surgery were reported. This could indicate that the presence of the virus (detected or unrecognized) is not a sufficient element to guarantee the risk of contagion of healthcare workers in the operating room, probably due to environmental conditions that are hostile to the spread of adequate viral loads or due to the presence of inactive viruses. This would mean that the presence of the virus in biological fluids, detected through a RT-PCR assay or not, does not reach the infecting viral load threshold.

Our last hypothesis is that the virus could not be always present. In a recent article, we hypothesized that the possible presence of SARS-CoV-2 in the peritoneum may depend on the disease stage and the associated cytokine storm [31]. In the severe form of COVID-19, the hyperinflammation, manifesting with cytokine storming, can lead to a greater viremic spread and, in such cases, it is possible that the virus would cross the pleural/peritoneal membrane. Moreover, in case of concomitant conditions able to increase the filtration gradient of the peritoneum (such as peritoneal dialysis or portal hypertension, with or without hepatic cirrhosis) it is possible that the virus would cross the membrane itself. As many other aspects of COVID-19, the involvement of biological fluids by SARSCoV-2 remains unexplored and only isolated reports dedicated to this topic were published.

Although the viral spread through the pleural and/or peritoneal fluid seems unlikely, there is urgent need for further evidence to confirm or refute this hypothesis, in order to help surgeons feel more comfortable and safe during their daily activities with COVID-19 inpatients.

## Conclusion

To date, there is no scientific evidence of possible contagion by laparoscopic aerosolization of SARS CoV- 2, nor it is known whether the virus is effectively present in biological fluids. However, it is important to consider that the methods employed in these studies to detect the presence of SARS-CoV-2, such as the variable number of samples, the different types of such tests and the immune status of patients can be confounding factors in the phase of viral detection. The possible relationship between higher viral loads (and consequently a greater viral burden in the pleural/peritoneal cavity) in symptomatic patients has not been clarified yet and, thus, needs further investigation. The impact of favoring open over minimally invasive techniques during the pandemic could be a health burden due to the prolonged length of hospitalization and cause of a higher rate of postoperative complications, precluding the gold standard approach (i.e., minimally invasive) for most patients.

On one hand, it is essential to provide healthcare workers with adequate PPE, especially in case of possible complications by unknown agents, and the general surgical community has been taking a cautious approach towards COVID-19 patients.

However, we also believe that depriving such patients of timely “gold standard” treatments, in favor of a cautious approach, such as the conservative management or the conventional open surgery, is not always the best choice for these patients and it might be matter of deeper collegial discussion.

## Supplementary Information

**Additional file 1: Table S1.** Body fluid swabs for Covid-19 in patients operated in Lagosanto Hospital, Ferrara, Italy. CCI: Charlson Comorbidity Index. 1st Wave: February 2020-July 2020; 2nd Wave: August 2020-December 2020; 3rd Wave: January 2021-today.

## Data Availability

The datasets generated during and analysed during the current study are not publicly available due to the rules and regulations of our hospital but are available from the corresponding author on reasonable request.
