# Supplementary material for: Swab test in biological fluids as predictor of COVID-19 transmission risk during surgery: a prospective cross-sectional study from an Italian COVID center
Source: BMC Surg. 2022 Mar 30;22:119. doi: 10.1186/s12893-022-01571-6 (PMC8964243; doi:10.1186/s12893-022-01571-6)
Supplement: Supplementary file 1 — Additional file 1: Table S1. Body fluid swabs for Covid-19 in patients operated in Lagosanto Hospital, Ferrara, Italy. CCI: Charlson Comorbidity Index. 1st Wave: February 2020-July 2020; 2nd Wave: August 2020-December 2020; 3rd Wave: January 2021-today. [file 12893_2022_1571_MOESM1_ESM.docx]

**Table S1.: Body fluid swabs for Covid-19 in patients operated in Lagosanto Hospital, Ferrara, Italy.** CCI: Charlson Comorbidity Index. 1^st^ Wave: February 2020-July 2020; 2^nd^ Wave: August 2020-December 2020; 3^rd^ Wave: January 2021-today.

|  | **Naso-pharyngeal Swab test** | **Age** | **Sex** | **CCI (points)** | **Covid-19 related pneumonia** | **Type of Swab** | **N. of samples** | **Result** | **Preop-NIV** | **1^st^ wave** | **2^nd^ wave** | **3^rd^ wave** | **In-hospital death** |
| --- | --- | --- | --- | --- | --- | --- | --- | --- | --- | --- | --- | --- | --- |
| **1** | Positive | 81 | F | 5 | Yes | Pleural | 1 | Negative | No | ✓ |  |  | Yes |
| **2** | Positive | 82 | F | 4 | Yes | Pleural | 1 | Negative | Yes |  |  | ✓ | no |
| **3** | Positive | 80 | M | 6 | Yes | Pleural | 3 | Negative | Yes |  | ✓ |  | Yes |
| **4** | Positive | 80 | F | 4 | Yes | Peritoneal | 3 | Negative | No | ✓ |  |  | Yes |
| **5** | Positive | 89 | F | 4 | Yes | Peritoneal | 3 | Negative | No |  |  | ✓ | No |
| **6** | Positive | 92 | M | 10 | Yes | Peritoneal | 2 | Negative | No |  |  | ✓ | Yes |
| **7** | Positive | 44 | F | 0 | No | Peritoneal and Biliary | 3 + 1 | Negative | No |  | ✓ |  | No |
| **8** | Positive | 74 | M | 3 | Yes | Peritoneal and Biliary | 2 +1 | Negative | Yes |  |  | ✓ | No |
